# 

**Published:** 1886-07

**Authors:** 


					﻿Whole Number,
CQEXII.
JillsY, 1886.
Number 12.
Volume XXVI.
The BUFFALO
iWwMgMlMWI
ESTABLISHED
IN YEAR 1844.
EDITORS $
THOS. LOTHROP, M. D.
A. R. DAVIDSON, M. D.
ASSOCIATE EDITORS:
FLOYD S. CREGO, M. D.
FRED. R. CAMPBELL, M. D.
CARLTON C. FREDERICK, M. D.
HERBERT MICKLE, M. D.
EDWARD B. ANGELL, M. D., Rochester, N. Y.
Entered at the Post-Office at Buffalo, N. Y., as Second-class Mail Matter.
				

